# HPLC/MS^n^ Profiling and Healing Activity of a Muco-Adhesive Formula of *Salvadora persica* against Acetic Acid-Induced Oral Ulcer in Rats

**DOI:** 10.3390/nu14010028

**Published:** 2021-12-22

**Authors:** Nahla Ayoub, Nadia Badr, Saeed S. Al-Ghamdi, Safaa Alsanosi, Abdullah R. Alzahrani, Ashraf B. Abdel-Naim, Khaled A. Nematallah, Noha Swilam

**Affiliations:** 1 Department of Pharmacology and Toxicology, Faculty of Medicine, Umm Al-Qura University (UQU), Makkah 21955, Saudi Arabia; sskghamdi@uqu.edu.sa (S.S.A.-G.); smsanosi@uqu.edu.sa (S.A.); aralzahrani@uqu.edu.sa (A.R.A.); 2 Saudi Toxicology Society, Umm Al-Qura University (UQU), Makkah 21955, Saudi Arabia; 3 Department of Dental Biomaterials, Faculty of Dentistry, Umm Al-Qura University (UQU), Makkah 21955, Saudi Arabia; nahassanein@uqu.edu.sa; 4 Institute of Cardiovascular & Medical Sciences, University of Glasgow, Glasgow G12 8QQ, UK; 5 Department of Pharmacology and Toxicology, Faculty of Pharmacy, King Abdulaziz University (KAU), Jeddah 21589, Saudi Arabia; aaabdulalrahman1@kau.edu.sa; 6 Department of Pharmacognosy, Faculty of Pharmacy, The British University in Egypt (BUE), El Sherouk City 11837, Egypt; khaled.nematallah@bue.edu.eg (K.A.N.); noha.swilam@bue.edu.eg (N.S.)

**Keywords:** *Salvadora persica* L., HPLC-ESI-QTOF-MS-MS analysis, anti-inflammatory, antioxidant, angiogenesis, oral ulcer, wound healing

## Abstract

*Salvadora persica* L. (*S. persica*, Siwak) is an ethnic plant that is widely used for improving oral hygiene. This study aimed to provide a phytochemical profiling of *S. persica* ethyl acetate fraction (SPEAF) and to evaluate the healing activity of a muco-adhesive formula of the fraction against acetic acid-induced oral ulcers in rats. HPLC-ESI-QTOF-MS-MS analysis of SPEAF resulted in the tentative identification of 56 metabolites containing fatty acids (23%), urea derivatives (10.5%) and sulphur compounds (10%), in addition to several amides, polyphenols and organic acids (6.5%, 5% and 2%, respectively). For the first time, 19 compounds were identified from *S. persica.* In vitro and in vivo experiments indicated that the extract is non-toxic. SPEAF exhibited superior healing activities compared to both the negative and positive control groups on days 7 and 14 of tongue ulcer induction. This was confirmed by histopathological examinations of haematoxylin and eosin-stained (H&E) and Masson’s trichrome-stained tongue sections. Moreover, SPEAF showed potent anti-inflammatory activities, as evidenced by the inhibited expression of interleukin-6 (IL-6) and tumour necrosis alpha (TNF-α). Moreover, SPEAF exhibited potent antioxidant activity, as it prevented malondialdehyde (MDA) accumulation, reduced glutathione (GSH) depletion and superoxide dismutase (SOD) exhaustion. SPEAF significantly enhanced hydroxyproline tongue content and upregulated collagen type I alpha 1 (Col1A1) mRNA expression. SPEAF also improved angiogenesis, as shown by the increased mRNA expression of the angiopoietin-1 (Ang-1). In conclusion, *S. persica* has a wide range of secondary metabolites and ameliorates acetic acid-induced tongue ulcers in rats. This can be attributed, at least partly, to its anti-inflammatory, antioxidant, procollagen and angiogenic activities. These findings provide support and validity for the use of *S. persica* as a traditional and conventional treatment for oral disorders.

## 1. Introduction

Several diseases of known and unknown aetiology can cause recurrent intraoral mucosal ulceration. Oral ulceration can be benign conditions, such as aphthae, herpetiform, erosive lichen planus, benign mucous membrane pemphigoid, allergy, infection, candidiasis, streptococcal stomatitis, histoplasmosis and acute necrotising ulcerative gingivitis, or conditions with a potential for a severe course, such as pemphigus vulgaris, lupus erythematosus, cyclic neutropenia neoplasm, Behcet’s disease and erythema multiforme [1,2].

Oral ulceration has been linked to a wide range of systemic medicines, with clinical manifestations ranging from superficial, nonspecific ulcerations to aphthous-like lesions or widespread mucosal erosions [3]. Nicorandil [4,5], captopril [6] and some nonsteroidal anti-inflammatory medicines can produce aphthous-like and nonspecific oral ulcers [7]. Oral ulcers can be classified as acute or chronic depending on their presentation and progress, with chronic ulcers lasting more than 14 days. [2]. 

Therapeutic options for the treatment of recurrent aphthous stomatitis include topical medications, antiseptics, analgesics, corticosteroids and antibiotics. In addition to systemic medication, immunomodulators have been reported [3]. Thalidomide is a tumour necrosis alpha (TNF-α) inhibitor with anti-inflammatory activity. However, due to teratogenic and other side effects (e.g., irreversible polyneuropathy), its use has been restricted [8]. The possible use of biological agents (e.g., infliximab) as anti-TNF-α therapy for recurrent aphthae in Behcet’s illness has also been documented [9].

Wound healing is a complex process involving haemostasis, inflammation, proliferation and tissue remodelling [10]. Many phyto-therapeutic studies have been conducted on oral antiulcerogenic activity based on haemostasis [11]; antioxidant [12,13,14], antibacterial [15] and anti-inflammatory activities [11,16,17,18,19]; proliferation/angiogenesis [20]; and remodelling phase/re-epithelialisation [11].

*Salvadora persica* L. Salvadoraceae (Siwak, *S. persica*) is a plant that has been used for many centuries as an oral hygiene tool, particularly in Saudi Arabia. Using Siwak (tooth stick) to clean the mouth is a well-established Islamic belief [21]. *S. persica* has been shown to have anti-gingivitis, anti-plaque and anti-cariogenic properties; gingival wound healing property; whitening capability; orthodontic chain preservation capability; and biocompatibility with oral cells in many investigations. *S. persica* in various forms has helped manage and treat oral health [22]. Comprehensive phytochemical investigations have revealed the presence of carbohydrates, flavonoids, terpenes, sterols, alkaloids, glycosides, organic sulphur compounds, elemental sulphur and small amounts of fluoride, calcium, phosphorus, silica and ascorbic acid [21,23].

Oral ulcers heal through the same phases as cutaneous wounds, but there are some differences, such as the wet external environment, the presence of saliva and various cell morphologies [24]. The environment of oral ulcers directs drug delivery to the application of muco-adhesive formulation [25,26]. In light of the research cited, this study prepared a fraction of *S. persica*, which has potential wound healing activity, in chemically induced tongue ulcers in rats and explored its components by using HPLC/MS/MS analysis.

## 2. Materials and Methods

### 2.1. Plant Material

*S. persica* sticks were bought from Riyadh, Saudi Arabia. The plant was identified by Dr. Talal Dahan, assistant professor of plant classification at Bisha University, Saudi Arabia. A voucher specimen (No. PG005) was deposited in properly labelled polythene bags for future reference at Department of Pharmacognosy, Faculty of Pharmacy, The British University in Egypt, Egypt. The authors followed the IUCN Policy Statement on Research Involving Species at Risk of Extinction.

### 2.2. Animals

Fifty male Wistar rats (200–225 g) were used for the study, courtesy of the Animal Facility, Faculty of Pharmacy, King Abdulaziz University (KAU), Saudi Arabia. The animals were kept on a 12 h light–dark cycle and a controlled temperature of 22 ± 2 °C. The research ethics committee of the Faculty of Pharmacy, King Abdulaziz University (KAU), officially approved experimental protocols (Reference # PH-1443-22).

### 2.3. Chemicals

All solvents were of analytical grade, while those used in UPLC/PDA/ESI/MS assays were of HPLC grade. Carboxymethyl cellulose (CMC) was obtained from Sigma-Aldrich (Taufkirchen, Germany).

### 2.4. Preparation of S. persica Ethyl Acetate Fraction (SPEAF)

Fresh plant sticks of *S. persica* were freeze-dried (laboratory freeze dryer VaCo 5, ZIRBUS, Bad Grund, Germany) and then ground to a fine powder using a commercially available food blender. A ground sample (500 g) was used to prepare the fraction. Ethyl acetate fraction was prepared by cold-percolating 500 g of dried powder of the plant sticks in 1 L of ethyl acetate for 72 h, and fresh solvent was used every 24 h. This fraction was prepared after isolating petroleum ether fraction using the same method described above. The solvent was removed and recovered in a rotary evaporator (Büchi Rotavapor RII; Büchi Labortechnik, Flawil, Switzerland) at 40 °C using a Büchi vacuum pump to yield 5.4 g of yellowish-brown powder, which was kept in a brown screw-capped tube in a −20 °C freezer until further analysis.

### 2.5. High-Resolution HPLC-ESI-QTOF-MS-MS Analysis

A 6530 QTOF LC/MS (Agilent Technologies) equipped with an autosampler (G7129A), a quat pump (G7104C) and a column comp (G7116A) was used for chromatographic separation. The injection volume was 6 μL. The analytes were separated in a Zorbax RP-18 column (Agilent Technologies; dimensions: 150 mm × 3 mm, dp = 2.7 μm) at a flow rate of 0.230 mL/min. The mobile phase consisted of a combination of solvent A (0.1% formic acid) and solvent B (acetonitrile + 0.1% formic acid). The gradient elution was as follows: 0–20 min (98–90% A), 20–50 min (90–80% A), 50–70 min (80–50% A), 70–90 min (50–30% A), 90–110 min (30–10% A) and 110–120 min (10–0% A) [27]. Mass spectra were simultaneously acquired using ESI in (+,−) ionisation modes, with a capillary voltage of 5500 V. The mass spectra were recorded in the m/z range of 100–1000 m/z. The gas temperature and drying gas flow were 190 °C and 6 L·/min, respectively. The skimmer and fragmentator voltages were set to 65 V and 130 V, respectively, and collision energy was 10 V. The nebulisation pressure was 25 psi g.

#### Tentative Identification of Metabolites

The tentative identification and analysis of LC-MS-MS of the metabolites of *S. persica* were carried out using Sirius^®^ software version 4.7.4 to predict fragmentation and molecular formulae [28]. The chemical structures were predicted using CSI: FingerID^®^ [29], while compound classes were predicted directly from MS/MS using CANOPUS^®^ [30].

### 2.6. Preparation of Plain and Ethyl Acetate Fraction Muco-Adhesive Formulae

In order to prepare the adhesive sponge formula, 2% CMC (*w*/*v*) was sprinkled in distilled water and stirred until homogenous gels formed, as prescribed by [25]. SPEAF was dissolved in distilled water to a final concentration of 5.0% (*w*/*v*) and stirred using a magnetic stirrer until homogeneous dispersions were achieved. Then, using a magnetic stirrer, CMC was sprinkled in the previously prepared dispersions until a consistent gel was achieved. Muco-adhesive property was achieved by the determination of muco-adhesive time of prepared formulae [25].

### 2.7. Cytotoxicity Assay

Oral epithelial cells (OEC) were obtained from Nawah Scientific Inc., (Mokatam, Cairo, Egypt). Cell viability was assessed by SRB assay. Aliquots of 100 μL cell suspension (5 × 103 cells) were pipetted in 96-well plates and incubated in complete media for 24 h. Cells were treated with another aliquot of 100 μL media containing SPEAF at various concentrations. After 72 h of exposure, cells were fixed by replacing media with 150 μL of 10% TCA and incubated at 4 °C for 1 h. The TCA solution was removed, and the cells were washed 5 times with distilled water. Aliquots of 70 μL SRB solution (0.4% *w*/*v*) were added and incubated in a dark place at room temperature for 10 min. Plates were washed 3 times with 1% acetic acid and allowed to air-dry overnight. Then, 150 μL of TRIS (10mM) was added to dissolve protein-bound SRB stains; the absorbance was measured at 540 nm by using a BMGLABTECH^®^-FLUOstar Omega microplate reader (Ortenberg, Germany) [31].

### 2.8. Acute Oral Toxicity Study

Acute oral toxicity of SPEAF was evaluated in male Wistar rats according to OECD guideline No. 423. Based on previous pilot studies in our laboratories, a limit test was performed. Animals were fasted overnight, and SPEAF was administered orally using gastric feeding needle at a dose of 2000 mg/kg [32].

### 2.9. Experimental Design

The animals were randomly divided into five groups, with 10 in each group. With the exception for negative control animals (10 rats), the rats were anaesthetized using ketamine (50 mg/kg) and xylazine (5 mg/kg). Round filter papers with a diameter of 5.0 mm were soaked in 15 mL of 50% acetic acid. The acid-soaked filter paper was pressed onto the inferior surface of the tongue for 60 s [33]. The animals were assigned into five groups, with 10 in each group, as follows.

Group 1 (negative control): normal rats with no exposure to acetic acid or any treatment.

Group 2 (acetic acid ulcer): acetic acid-challenged animals with no treatment.

Group 3 (ulcer + vehicle): acetic acid ulcer group treated topically once daily with plain muco-adhesive formulae in the ulcer area.

Group 4 (ulcer + SPEAF): acetic acid ulcer group treated topically once daily with SPEAF (5% in muco-adhesive formulae) in the ulcer area.

Group 5 (positive control): acetic acid ulcer group treated topically once daily with Jogel^®^ (Sedico, Giza, Egypt; 10% jojoba oil and 0.5% lidocaine hydrochloride) in the ulcer area. 

All treatments continued for 14 days. On day seven, four animals from each group were sacrificed by decapitation, and their tongues were dissected and kept in 10% neutral formalin. On day 14, the rest of the animals in all groups were sacrificed by decapitation, and their tongues were dissected. One part of the tongues from each animal was kept in 10% neutral formalin, and the other part was flash frozen in liquid nitrogen and kept at −80 °C for further analysis.

### 2.10. Histopathological Examination

The excised tongue tissues on days 7 and 14 were fixed in 10% neutral buffered formalin. They were dehydrated in serial dilutions of ethyl alcohol, immersed in xylene and embedded in paraffin. On the glass slides, 5-micrometre-thick sections were made. After dewaxing, the tissue sections were rehydrated. Some sections were stained using haematoxylin and eosin (H&E), and others were stained with Masson’s trichrome stains.

### 2.11. Immunohistochemical Staining

The tissue sections were de-paraffinised, rehydrated and boiled in 0.1 M citrate buffer (pH 6.0) for 10 min. The sections were then kept in 5% bovine serum albumin in Tris-buffered saline (TBS) for 2 h. The tissue sections were then incubated with primary antibodies to interleukin-6 (IL-6), (Cat. No.: ab9324, Abcam^®^, Cambridge, UK) and TNF-α (Cat. No.: ab220210, Abcam^®^, Cambridge, UK) at 4 °C for 12 h. Following flushing with TBS, the tissue sections were incubated with either anti-mouse or anti-rabbit biotinylated secondary antibody according to the primary antibody reactivity (Cell & Tissue Staining Kit, Cat. No.: CTS002, CTS006, R&D Systems, Minneapolis, MN, USA). Image evaluations were obtained using Image J (1.52a, National Institutes of Health NIH, Rockville, Maryland, USA) with a minimum of three sections per rat.

### 2.12. Biochemical Analysis

The tongue tissues were homogenised in a 10-fold volume of ice-cooled phosphate-buffered saline (50 mM potassium phosphate, pH 7.4). The homogenates were centrifuged for 15 min at 10,000× *g* and 4 °C, followed by the collection of the supernatant, which was used for oxidative stress analysis. Commercially available kits were used to assess the liver content of malondialdehyde (MDA; Cat. No. MD 2529; Biodiagnostic, Giza, Egypt) and reduced glutathione (GSH; Cat. No. GR 2511; Biodiagnostic, Giza, Egypt) and the enzyme activities of superoxide dismutase (SOD; Cat. No. SD 2521; Biodiagnostic, Giza, Egypt). Hydroxyproline was determined using ELISA kits (Cat. No. Ab22294, Abcam, Cambridge, UK), according to the manufacturer’s instructions.

### 2.13. RT-qPCR for Collagen Type I Alpha 1 (Col1A1) and Angiopoietin-1 (Ang-1)

Tongue homogenates were subjected to RNA extraction by using a commercially available kit (NucleoSpin, Macherey-Nagel GmbH & Co. KG, Duerin, Germany), followed by spectrophotometric determination of purity and concentration (dual-wavelength Beckman, Spectrophotometer, Foster City, California, USA). cDNA was performed using a cDNA Reverse Transcription Kit (Applied Biosystems, Foster City, CA, USA). Then, a PCR Master Mix Kit (Qiagen, Valencia, CA, USA) was used to perform PCR amplification reactions. The Col1A1 (NM_053304.1) primer and GAPDH (NM_017008.4) as housekeeping genes were used. The forward/reverse nucleotide sequences of Col1A1, Ang-1 and GAPDH were 5′-ATCAGCCCAAACCCCAAGGAGA-3/5′-CGCAGGAAGGTCAGCTGGATAG-3, 5′-AGGCCCCTCTGAACCCTAAG-3/5′-AGAGGCATACAGGGACAACACA-3 and 5′-CCATTCTTCCACCTTTGATGCT-3/5′-TGTTGCTGTAGCCATATTCATTGT-3, respectively. The data were expressed in the cycle threshold (Ct). The expression of Col1A1 relative to GAPDH was calculated based on the delta–delta Ct (ΔΔCt) values.

### 2.14. Statistical Analysis

All data were expressed as mean ± SD. The data were analysed using a one-way analysis of variance and Tukey test. All analyses were performed using GraphPad Prism software version 8.00 (GraphPad Software, La Jolla, CA, USA). A *p* value of less than 0.05 was considered statistically significant.

## 3. Results

### 3.1. Identification of SPEAF

For the complete phytochemical profiling of the ethyl acetate fraction, a high-resolution ESI-QTOF-LC-MS-MS analysis was carried out. A total of 56 compounds were detected and tentatively identified (Table 1), 19 of which were identified in *S. persica* for the first time. The majority of the compounds identified were fatty acids (23%), followed by urea derivatives (10.5%) and sulphur compounds (10%). Several amides, phenolic compounds and organic acids were also detected at 6.5%, 5% and 2%, respectively. Other individual minor compounds were detected at 1.5%.

Fifteen different fatty acids were tentatively identified. Palmitic and oleic acids were the major fatty acids detected; both were identified previously in the stems and roots of *S. persica* [34]. Myristic acid (36), hydroxy octadecenoic acid (37), hexadecenoic acid (3), arachidic acid (40), linoleic acid (41), heptadecenoic acid (44) and stearic acid (54) were detected previously in the stems and roots of *S. persica* [23,34], while linolenic acid (35) was detected previously in the seed oil of *S. persica* [35]. Hydroxytetradecanoic acid (33), hydroxyhexadecanoic acid (34), hydroxyoctadecanoic acid (45), nonadecenoic acid (51) and hydroxyeicosanoic acid (53) were detected in this plant for the first time.

Three urea derivatives were detected in the fraction, and the majority of them consisted of N,N′-dibenzyl urea (30, 7.7%), followed by benzyl urea (13, 2.5%) and n-benzyl-N′ hydroxy benzyl urea (25, 0.09%). These compounds were detected and isolated previously in this species [23,36].

Four sulphur-containing compounds were also detected. Benzyl isothiocyanate (18) was the major compound found in the fraction (8.7%); it was detected previously with *O*-benzyl hexosyl sulphate (14) in *S. persica* [23,34]. The molecular formulae of the sulphated hexosyl phenolic derivative (10) and sulphur compound derivative (32) are C_14_H_20_O_11_S and C_12_H_12_N_2_O_2_S_2_, respectively. Both compounds were not detected before in genus *Salvadora*.

Ten amide derivatives were also observed, most of which were amide derivatives of fatty acids. Compounds *n*-benzyl benzamide (28), *n*-benzyl 2-phenyl acetamide (29), *n*-benzylpalmitamide (49) and *n*-benzyl heptadecanamide (52) were identified previously in *S. persica* [23,36], while benzamide (7), methylbenzamide (11), fatty acid amide derivatives (42, 43), *n*-benzyl octadecenamide (50) and 13-docosenamide (56) were detected in this plant for the first time.

Regarding phenolic compounds, 11 compounds were detected. All were phenolic acids and their derivatives, except compound 23 (methoxy flavanone hexosyl rhamnoside), which is a flavonoid glycoside. With a molecular formula of C_15_H_22_O_5_, compound 16 was detected for the first time in *S. persica*, and it was tentatively identified as a phenolic acid derivative. All other phenolic compounds were previously reported in the same plant [23,37,38,39]. Three organic acids and their derivatives were identified. Salicylic and glutaric acids were detected previously in the plant [37,40], whereas compound 9 (diethyl malate) was detected for the first time in *S. persica.*

### 3.2. IC50 of SPEAF on OEC

**Figure 1:**
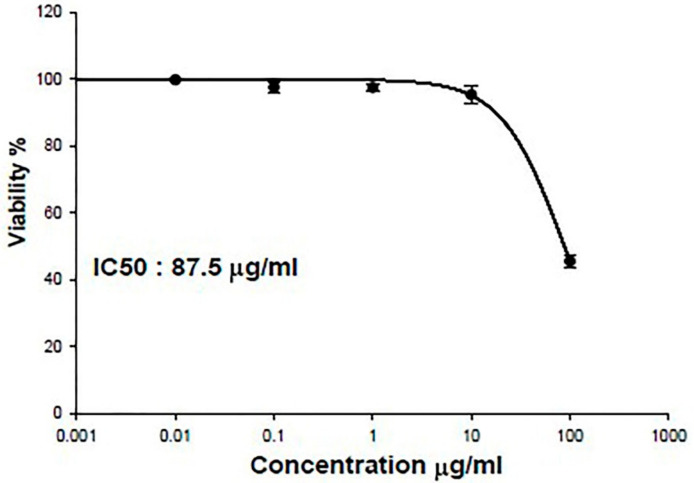
IC50 of SPEAF in oral epithelial cells (OEC).

As shown in Figure 1, IC50 of SPEAF in oral epithelial cells (OEC) was relatively high (87.5 μg/mL) indicating very weak cytotoxicity of the extract.

### 3.3. Acute Oral Toxicity Study

No mortality was observed in the tested animals at 24 h after oral administration of 2000 mg/kg of SPEAF. The same result was obtained when the test was repeated using three additional animals at the same dose. According to the Acute Toxic Class Method reported in OECD guidelines No.423, SPEAF is considered to be Category 5 with LD50 > 2000 mg/kg.

### 3.4. Histopathological Examination

**Figure 2:**
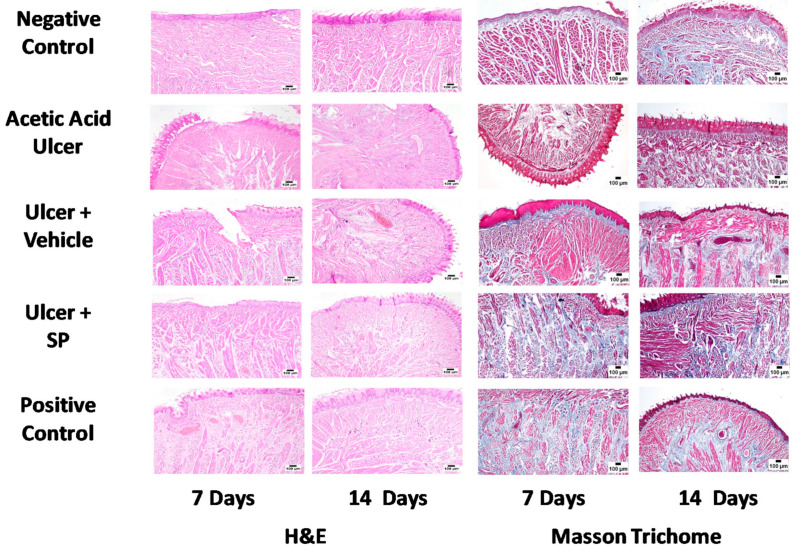
Histopathological effects of SPEAF on acetic acid-induced tongue ulcer of rats. SPEAF: *S. persica* ethyl acetate fraction. Haematoxylin and Eosin (H&E).

Microscopic examination of H&E-stained tongue sections from negative control animals on days 7 and 14 showed skeletal muscle arranged in longitudinal and transverse bundles with a normal histological structure. The tongues were covered by papillae and stratified squamous epithelium that appeared keratinised on the dorsal surface. The acetic acid group showed several ulcerative mucosal surfaces, with necrotic areas that extended to the underlying muscle bundles. Severely dilated blood spaces were commonly observed. Vehicle-treated animals exhibited ulceration of the epithelial covering layer accompanied by variable haemorrhages and the accumulation of eosinophilic tissue debris. Dispersions of muscle bundles with oedema, inflammatory cells and increased number of mast cells were also observed. SPEAF-treated animals showed obvious protection against acetic acid ulcers. The mucosal surface appeared normal in several examined sections. A few sections showed ulcerative epithelial layers, while other sections revealed an aggregation of perivascular inflammatory cell infiltration. Positive control animals exhibited noticeable protection, as numerous sections showed marked healing of the ulcerative area, but congested blood vessels with ulceration were observed in some sections. In order to substantiate these observations, collagen deposition was assessed by staining sections from all groups using Mason’s trichrome stain. The negative control animals showed normal histological architecture. The acetic acid group showed tongue sections with a relatively decreased deposition of collagen fibers. The tongue sections from the SPEAF and positive control groups exhibited a higher degree of collagen deposition (Figure 2).

### 3.5. Immunohistochemical Assessment of the Expression of Inflammation Markers

**Figure 3:**
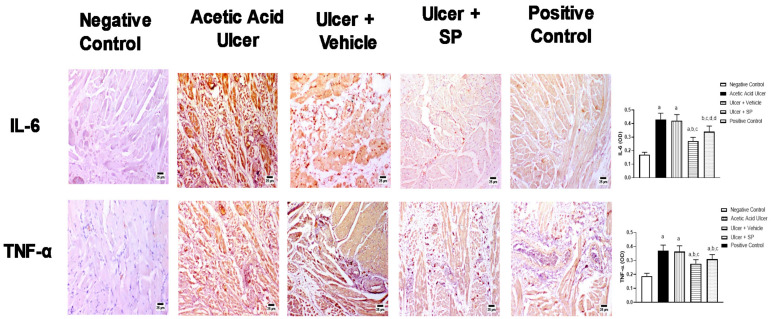
Effect of SPEAF on expression of inflammation markers in acetic acid-induced tongue ulcer in rats. Statistical analysis was performed by one-way ANOVA followed by Tukey’s test. ^a^ Significant difference from negative control group at *p* < 0.05. ^b^ Significant difference from acetic acid group at *p* < 0.05. ^c^ Significant difference from ulcer + vehicle group at *p* < 0.05. ^d^ Significant difference from Ulcer + SPEAF group at *p* < 0.05. SPEAF: *S. persica* ethyl acetate fraction.

The potential of SPEAF as an anti-inflammatory agent was assessed in stressed tongue tissues. Figure 3 shows that the challenge in tongues with acetic acid was associated with the enhanced expressions of IL-6 and TNF-α by 176% and 84%, respectively, compared to corresponding control values. The application of SPEAF significantly reduced IL-6 and TNF-α expressions by 36% and 24%, respectively, compared to the acetic acid-alone group. Similarly, the treatment of tongues with the positive control preparation significantly inhibited IL-6 and TNF-α by 20% and 15%, respectively, compared to the acetic acid ulcer group.

### 3.6. Assessment of Oxidative Status

**Figure 4:**
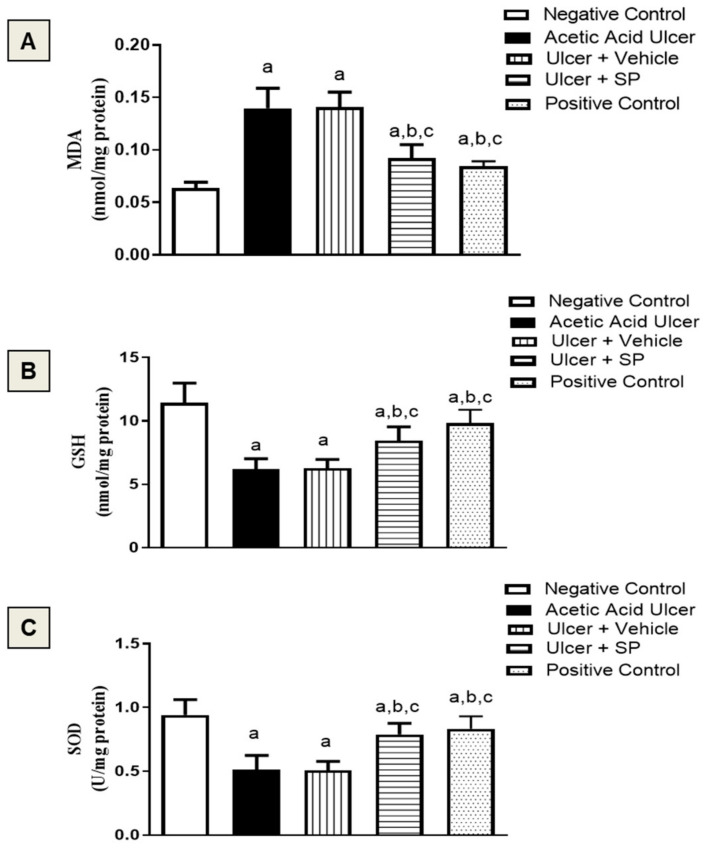
Effect of SPEAF on MDA (**A**), GSH (**B**) and SOD (**C**) in acetic acid-induced tongue ulcer in rats. Data are presented as mean ± SD (*n* = 6). Statistical analysis was performed by one-way ANOVA followed by Tukey’s test. ^a^ Significant difference from negative control group at *p* < 0.05. ^b^ Significant difference from acetic acid group at *p* < 0.05. ^c^ Significant difference from ulcer + vehicle group at *p* < 0.05. SPEAF: *S. persica* ethyl acetate fraction. MDA: malondialdehyde. GSH: glutathione. SOD: superoxide dismutase.

Figure 4A shows that MDA, a product of polyunsaturated fatty acid peroxidation, was increased by an acetic acid challenge by 133% of the control value. SPEAF ameliorated MDA accumulation by 36%. Moreover, acetic acid resulted in GSH depletion compared to the negative control values. SPEAF and the positive control preparation significantly ameliorated GSH decrease by 34% and 56%, respectively, compared to the acetic acid group (Figure 4B). Acetic acid insult was associated with decreased SOD activity. Both SPEAF and the positive control preparation inhibited SOD exhaustion by 60% and 62%, respectively (Figure 4C).

### 3.7. Assessment of Collagen Content

**Figure 5:**
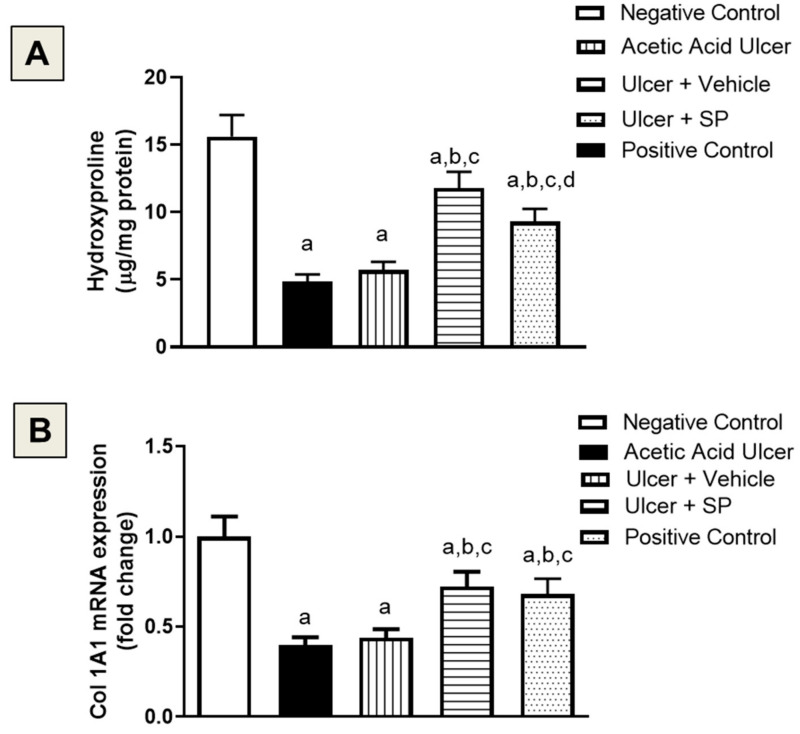
Effect of SPEAF on hydroxyproline content (**A**) and Col1A1 expression (**B**) in acetic acid-induced tongue ulcer in rats. Data are presented as Mean ± SD (*n* = 6). Statistical analysis was performed by one-way ANOVA followed by Tukey’s test. ^a^ Significant difference from negative control group at *p* < 0.05. ^b^ Significant difference from acetic acid group at *p* < 0.05. ^c^ Significant difference from ulcer + vehicle group at *p* < 0.05. ^d^ Significant difference from ulcer + SPEAF group at *p* < 0.05. SPEAF: *S. persica* ethyl acetate fraction.

Acetic acid-induced tongue ulcers were associated with a significant decrease in hydroxyproline by 57% compared to the negative control group. The SPEAF group showed the highest hydroxyproline content, as it was significantly enhanced by 76% compared to the acetic acid group (Figure 5A). These results were confirmed by assessing the mRNA expression of Col1A1. Both SPEAF and the positive control preparation significantly enhanced Col1A1 expression by 64% and 55%, respectively, compared to the acetic acid group (Figure 5B).

### 3.8. Assessment of Ang-1 mRNA Expression

**Figure 6:**
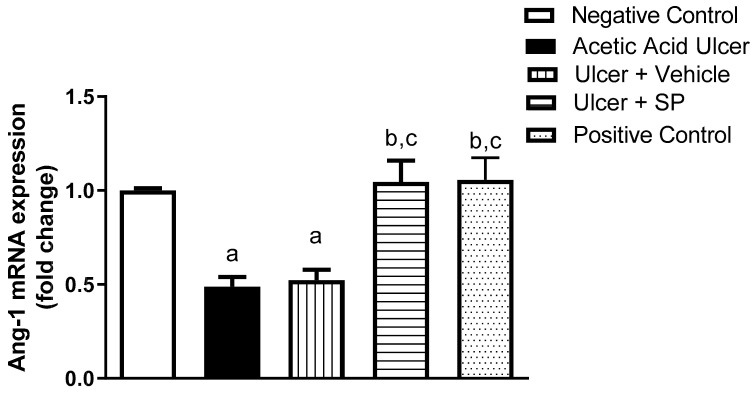
Effect of SPEAF on Ang-1 mRNA expression in acetic acid-induced tongue ulcer in rats. Data are presented as mean ± SD (*n* = 6). Statistical analysis was performed by one-way ANOVA followed by Tukey’s test. ^a^ Significant difference from negative control group at *p* < 0.05. ^b^ Significant difference from acetic acid group at *p* < 0.05. ^c^ Significant difference from ulcer + Vehicle group at *p* < 0.05. SPEAF: *S. persica* ethyl acetate fraction.

The potential angiogenic activity of SPEAF was assessed in the tongue tissues. Figure 6 shows that SPEAF, and positive control preparation significantly enhanced Ang-1 mRNA expression by an approximately one-fold increase compared to the acetic acid group.

## 4. Discussion and Conclusions

Oral ulcers are a common disorder of the oral mucosa. There are many predisposing conditions, including systemic diseases [41]. The primary treatment of oral ulcers includes the use of local corticosteroids in addition to antiseptic/anti-inflammatory agents and local anaesthetics [42]. However, these may have intolerable adverse effects [42,43]. For this reason, natural products have attracted great attention, as they constituted 16% of FDA-approved drugs in 2018 [44]. In particular, *S. persica* is considered to be nature’s gift for oral health [45,46]. The current study presents a phytochemical profile of SPEAF using HPLC-ESI-QTOF-MS-MS analysis. Nineteen compounds were detected for the first time in this plant, 12 of which were tentatively identified as benzamide (7); diethyl malate (9); methylbenzamide (11); acetyl phenylalanine (20); hydroxytetradecanoic acid (37); hydroxyhexadecanoic acid (34); hydroxyoctadecanoic acid (45); n-benzyl octadecenamide (50); nonadecanoic acid (51); hydroxyeicosanoic acid (53); diisooctyl phthalate (55); and 13-docosenamide (56). According to Sirius, compounds 5 and 16 were a sugar derivative and a phenolic acid derivative, with molecular formulae of C_10_H_20_O_8_ and C_15_H_22_O_5_, respectively. Compounds 10 and 32 were sulphated compound derivatives, with molecular formulae of C_14_H_20_O_11_S and C_12_H_12_N_2_O_2_S_2_, respectively. Compounds 42 (C_21_H_35_NO) and 43 (C_23_H_37_NO) were fatty acid amide derivatives, while compound 47 was a cholesterol derivative, with the molecular formula C_39_H_58_N_4_O_5_. Moreover, the healing activity of a muco-adhesive formula of *S. persica* against acetic acid-induced oral ulcers in rats was evaluated for the first time. Our data highlighted the safety of the extract. In addition, it expedited healing rates in wounded animals. This was confirmed histologically, as microscopic examinations indicated an almost intact epithelial covering, with fewer signs of inflammation and enhanced collagen deposition. This is consistent with previously published experimental data on the wound-healing potential of the plant in the skin or the tongue [47,48].

The transition from the inflammatory phase to the proliferative phase is a key step during wound healing [49]. Consequently, if the inflammatory response is extended or exacerbated, it results in a delay in the subsequent phases of proper wound healing [49,50]. Our results showed that the observed healing activity of *S. persica* was accompanied by a shortening of the inflammation phase. This was highlighted by the ability of *S. persica* in animal models of peptic ulcer [51] and paw oedema [52]. The effectiveness of *S. persica* gel application in the treatment of moderate and deep pockets in patients with periodontitis was associated with the inhibition of the crevicular fluid IL-6 and TNF-α [52,53]. Flavonoids have been reported to possess potent anti-inflammatory activity [54]. Based on our phytochemical profiling study, it can be suggested that the *S. persica* content of gallic acid (10), deoxy ellagic acid (3), methoxy ellagic acid (6), sulphated hexosyl polyphenols (10), *O*-benzyl hexosyl sulphate (14), phenolic acid derivatives (16, C_15_H_22_O_5_), di-*O*-methyl ellagic acid (17), coumaric acid (19), ferulic acid (22), methoxy flavanone hexosyl rhamnoside (23) and caffeic acid conjugate (24), representing 5% of the identified compounds of SPEAF, may contribute to observed anti-inflammatory activities. In addition, the major fatty acids identified were palmitic acid (4.98%), which has anti-inflammatory effects on endothelial cells (ECs) with TNF-α and counter endothelial dysfunction [55] and oleic acids (4.71%), which is an anti-inflammatory fatty acid that plays a role in the activation of different pathways of immune-competent cells [56]. The identified urea derivatives (10.5%, with N,N′-dibenzyl urea; 7.7%) showed promising anti-inflammatory activity (62–84% TNF-α and 73–92% IL-6 inhibitory activity) at a concentration of 10 μM, with reference to standard dexamethasone [57]. Moreover, in addition from having a reported potent bactericidal activity against oral pathogens and MRSA [58], the detected sulphur compound, benzyl isothiocyanate (8.7%), significantly attenuated TPA-enhanced hydrogen peroxide levels and acted as an inhibitor of O2 generation in mouse skin [59].

Oxidative stress contributes to the pathology of ulcerogenesis. Conversely, antioxidants reduce cellular damages that arise as a consequence of the increased generation of oxidative species [60]. The present study highlights the antioxidant activity of *S. persica* in tongue tissues and is supported by several studies [61,62,63]. Based on our phytochemical analyses of *S. persica* extract, antioxidant activity can be attributed to its polyphenol (5%) [64] and fatty acid (23%) content [65]. Farag et al. (2018) examined the antioxidant activities of *S. persica* ethanol extract and some isolated compounds called persicaline [66]. Essentially, antioxidants have been reported to play an important role in expediting wound healing by reducing oral mucosa inflammation, thus decreasing the risk of developing precancerous lesions [67]. This provides further insight into the healing properties of *S. persica*. This also supports observed anti-inflammation, as oxidative stress and inflammation are interconnected. The generated ROS induces the activation of transcription factors, which drive the expression of pro-inflammatory mediators, such as IL-6 and TGF-β [68].

Collagen, a key protein of the extracellular matrix produced by fibroblasts, is involved in the healing of connective tissues [69]. The final stages of wound healing involve remodelling, which should result in the closure of wounds or ulcers. In this context, collagen and hydroxyproline play an important role [70]. The results from this study indicated increased hydroxyproline formation and Col1A1 mRNA expression in the *S. persica*-treated group in comparison to untreated animals. In accordance with these findings, *S. persica* extract has been reported to inhibit collagen degradation in demineralised dentin [71]. Moreover, *S. persica* extract-laden collagen hybrid constructs showed enhanced periodontal tissue regeneration [72], consistent with the ability of *S. persica* to enhance fibroblast proliferation and viability [73]. This also supports the observed ability of *S. persica* to enhance angiogenesis, as evidenced by increased Ang-1 mRNA expression. In other words, collagen deposition in wound tissues is associated with enhanced angiogenesis [74]. Angiogenesis plays a key role in the wound-healing process and is involved in the migration, growth and differentiation of ECs [75]. The ability of naturally occurring compounds has been reviewed [76]. Our data are supported by the ability of flavonoids to boost angiogenesis in non-cancerous tissues [77]. In conclusion, *S. persica* has a wide range of secondary metabolites ameliorate acetic acid-induced tongue ulcers in rats. This can be attributed, at least partly, to its anti-inflammatory, antioxidant, procollagen and angiogenic activities. These findings provide support and validity for the use of *S. persica* as a traditional and conventional treatment for oral disorders and validate its application in the treatment of oral ulcers, which warrants further clinical studies.

## Figures and Tables

**Table 1:** LC–MS–MS data of the tentatively identified compounds in SPEAF.

#	Retention Time	Compound	Area%	MS1 (−ve)	MS1 (+ve)	MS2	Molecular Formula	Error
1	4.02	Gallic acid	1.5	169.01417		125	C_7_H_6_O_5_	6.04
2	10.6	Glutaric acid	0.43	131.042		---	C_5_H_8_O_4_	−1.53
3	11.16	Deoxy ellagic acid	0.67	287.0191		241, 181, 151	C_14_H_8_O_7_	−2.08
4	13.97	Hydroxy stachrydine	tr	158.0822		141, 131, 115	C_7_H_13_O_3_N	−0.6
5	16.17	Sugar derivative	0.43	267.1081		221, 153	C_10_H_20_O_8_	−0.41
6	21.2	Methoxy ellagic acid	0.3	315.01459		241, 181, 151	C_15_H_8_O_8_	−0.16
7	22.38	Benzamide	1.13		122.0599	105	C_7_H_7_NO	−1.15
8	23.95	Salicylic acid	0.73	137.0231		---	C_7_H_6_O_3_	−1.28
9	24.69	Diethyl malate	0.7	189.0764		145, 100	C_8_H_14_O_5_	−0.46
10	25.4	Sulfated hexosyl phenolic derivative	0.22	395.0676		315, 241, 153	C_14_H_20_O_11_S	2.22
11	27.067	Methylbenzamide	0.32		136.0747	---	C_8_H_9_NO	−7.27
12	27.41	Unknown	1.43	194.0809		164, 134	C_10_H_13_NO_3_	−1.31
13	28.111	Benzyl urea	2.52		151.0857	---	C_8_H_10_N_2_O	5.89
14	31.31	O-benzyl hexosyl sulfate	0.42	349.0585		269, 241, 193	C_13_H_18_O_9_S	−3.94
15	32.83	Unknown	0.35	521.2331 (2M − H)		260	C_11_H_19_NO_6_	−2.97
16	34.32	Phenolic acid derivative	0.14	281.13909		151	C_15_H_22_O_5_	−1.27
17	34.36	Di-O-methyl ellagic acid	0.98	329.032		315, 241, 181, 151	C_16_H_10_O_8_	5.19
18	35.902	Benzyl isothiocyanate	8.7		150.0374	---	C_8_H_7_NS	−5.95
19	37.29	Coumaric acid	tr	163.03897		119	C_9_H_8_O_3_	−1.54
20	38.88	Acetyl Phenyl alanine	tr	413.16868 (2M − H)	415.1810 (2M + H)	206, 188	C_11_H_13_NO_3_	−7.58
21	47.645	Unknown	1.53		123.0431	---	C_5_H_4_N_3_O	3.15
22	56.8	Ferulic acid	0.39	193.0492		179, 149	C_10_H_10_O_4_	−1.74
23	57.55	Methoxy flavanone hexosyl rhamnoside	0.58	609.18129		463, 301	C_28_H_34_O_15_	−1.98
24	59.78	Caffeic acid conjugate	0.13	387.0352		341, 193	C_18_H_12_O_10_	−3.41
25	60.399	N-benzyl-N′ hydroxy benzyl urea	0.09		257.1269	241, 198, 181, 163	C_8_H_10_N_2_O_2_	−6.03
26	66.42	Caffeic acid conjugate	0.07	377.18179		341, 161	C_17_H_30_O_9_	1.67
27	67.61	Syringin	0.15	371.1344		209	C_17_H_24_O_9_	0.51
28	70.88	N-benzyl benzamide	0.15		212.1057	---	C_14_H_13_NO	−6.08
29	71.663	N-benzyl 2-phenyl acetamide	0.2		226.1215	---	C_15_H_15_NO	−5.04
30	71.7	N,N′ dibenzyl urea	7.73		241.1326	181, 163, 108	C_15_H_18_N_2_O	−3.9
31	74.819	Unknown	0.06		353.1957	---	C_19_H_28_O_6_	−1.94
32	75.37	Sulfur compound derivative	0.61		281.0402	186	C_12_H_12_N_2_O_2_S_2_	−7.8
33	92.482	Hydroxy tetradecanoic acid	3.94	487.4005 (2M − H)		243	C_14_H_28_O_3_	1.31
34	102.79	Hydroxy hexadecanoic acid	0.69	543.4565 (2M − H)		271.2266	C_16_H_32_O_3_	−0.63
35	106.09	Linolenic acid	1.49	555.4408 (2M − H)	557.4496 (2M + H)	277	C_18_H_30_O_2_	−2.01
36	107.327	Myristic acid	0.45	455.4111 (2M − H)		227	C_14_H_28_O_2_	1.19
37	108.559	Hydroxy octadecenoic acid	0.99	595.4890 (2M − H)		297	C_18_H_34_O_3_	−8.92
38	109.176	Hexadecenoic acid	0.95	507.4416 (2M − H)	509.4512 (2M + H)	253	C_16_H_30_O_2_	−0.64
39	109.35	Unknown	3.09	339.2299		253, 113	C_23_H_32_O_2_	−3.05
40	111.332	Arachidic acid	0.12		313.2727	285, 267	C_20_H_42_O_2_	4.52
41	111.527	Linoleic acid	2.43	559.4781 (2M − H)	561.4821 (2M + H)	279	C_18_H_32_O_2_	−5.52
42	111.552	Fatty acid amide derivative	0.45		635.5489 (2M + H)	318	C_21_H_35_NO	−1.89
43	113.309	Fatty acid amide derivative	0.88		687.5803 (2M + H)	344	C_23_H_37_NO	−4.5
44	113.598	Heptadecenoic acid	0.29	535.4730 (2M − H)	537.4845 (2M + H)	267	C_17_H_32_O_2_	−0.36
45	115.622	Hydroxy octadecanoic acid	0.54	599.5241 (2M − H)		299	C_18_H_36_O_3_	−2.59
46	116.188	Palmitic acid	4.98	511.4714 (2M − H)	513.4861 (2M + H)	255	C_16_H_32_O_2_	−2.42
47	116.23	Cholesterol derivative	tr		663.4529	607, 551	C_39_H_58_N_4_O_5_	5.74
48	117.307	Oleic acid	4.71	563.5026 (2M − H)	565.5175 (2M + H)	281	C_18_H_34_O_2_	−2.38
49	118.394	N-benzylpalmitamide	0.06		691.6144 (2M + H)	346	C_23_H_39_NO	−0.42
50	120.34	N-benzyl octadecenamide	1.79		743.6433 (2M + H)	372	C_25_H_41_NO	−0.05
51	121.418	Nonadecenoic acid	0.26	591.5330 (2M − H)		295	C_19_H_36_O_2_	−3.79
52	122.592	N-benzyl heptadecanamide	0.1		719.6405 (2M + H)	360	C_24_H_41_NO	3.63
53	123.163	Hydroxy eicosanoic acid	0.73	655.5859 (2M − H)		327	C_20_H_40_O_3_	−2.7
54	123.505	Stearic acid	0.31	567.5334 (2M − H)		283	C_18_H_36_O_2_	−3.24
55	123.666	Diisooctyl phthalate	1.2		391.2832	167, 149	C_24_H_38_O_4_	−5.58
56	125.314	13-Docosenamide	1.44		338.3416	321	C_22_H_43_NO	−0.13

## Data Availability

The data that support the findings of this study are openly available from the corresponding author upon reasonable request.
